# Chinese herbal prescriptions for COVID-19 management: Special reference to Taiwan Chingguan Yihau (NRICM101)

**DOI:** 10.3389/fphar.2022.928106

**Published:** 2022-10-05

**Authors:** Yi-Chang Su, Guan-Jhong Huang, Jaung-Geng Lin

**Affiliations:** ^1^ National Research Institute of Chinese Medicine, Ministry of Health and Welfare, Taipei, Taiwan; ^2^ Department of Chinese Pharmaceutical Sciences and Chinese Medicine Resources, College of Chinese Medicine, China Medical University, Taichung, Taiwan; ^3^ Department of Food Nutrition and Healthy Biotechnology, Asia University, Taichung, Taiwan; ^4^ School of Chinese Medicine, College of Chinese Medicine, China Medical University, Taichung, Taiwan

**Keywords:** SARS-CoV-2, antivirus, anti-inflammation, traditional Chinese medicine, viral infection

## Abstract

Severe acute respiratory syndrome coronavirus 2 (SARS-CoV-2) is a strain of coronavirus that causes COVID-19 (coronavirus disease 2019), the respiratory illness responsible for the ongoing COVID-19 pandemic. As at June 2022, increasing numbers of newly diagnosed COVID-19-associated pneumonia cases worldwide have attracted close attention from the international community. The present review analyzes and summarizes the treatment of COVID-19 with traditional Chinese medicine (TCM). A systematic analysis of the efficacies and benefits of TCM for the treatment of COVID-19 was performed, and the mechanisms underlying such treatment are summarized. This analysis of the literature highlights the potential of TCM to prevent and treat COVID-19 *via* antiviral, anti-inflammatory and immunomodulatory activities, with evidence showing that many TCM components act upon multiple targets and pathways. Famous TCM formulas include Qing-Fei-Pai-Du-Tang (QFPDT), Lianhuaqingwen Capsule (LHC), Taiwan Chingguan Yihau (NRICM101), and Jing Si herbal drink (JSHD). In particular, the botanical preparation NRICM101 was developed in 2020 for use in viral respiratory tract infections and is recommended for treating non-severe and mild COVID-19 infections. NRICM101 has been adopted for use in Taiwan for the clinical treatment of COVID-19. The common components and active ingredients of 10 TCM preparations have been analyzed for the most promising substances. This review aims to provide reliable evidence demonstrating the therapeutic efficacy of TCM substances in support of their further development against novel coronavirus infectious diseases in Taiwan.

## 1 Introduction

Since its emergence in December 2019, severe acute respiratory syndrome coronavirus 2 (SARS-CoV-2, COVID-19) has had devastating effects worldwide, with more than 362 million confirmed cases and 5.62 million deaths globally by early February 2022 (Wu et al., 2020a). As at 14 June 2022, epidemic real-time data had recorded 300,501 COVID-19 diagnoses and 4,403 deaths in Taiwan, involving 28,192 immigrants and 2,975,309 native cases (MOHW, 2022). The process of COVID-19 infection involves angiotensin-converting enzyme 2 (ACE2), transmembrane protease serine-2 (TMPRSS2) and 3CL protease (3CL^pro^) (Hoffmann et al., 2020; Hsu et al., 2020). By binding to the ACE2 receptor, the SARS-CoV-2 spike protein (S protein) drives viral reproduction (Ni et al., 2020), entering host cells *via* the major host protease, TMPRSS2 (Shang et al., 2020), while 3CL^pro^ is the main protease of SARS-CoV-2 and responsible for viral replication, so is considered to be an important drug target (Mody et al., 2021; Niesor et al., 2021; Wang and Yang, 2021a; Wang et al., 2021).

The clinical symptoms of COVID-19 are most commonly fever, cough, fatigue, myalgia, diarrhea and complications arising from multiple organ damage (Zaim et al., 2020). None of these symptoms are as yet treatable by small-molecule antivirals. Effective prevention and treatment measures are critical for the control of COVID-19 (Jiang et al., 2020).

Traditional Chinese medicine (TCM) philosophy categorizes COVID-19 as an “epidemic disease” caused by an epidemic evil with dampness and heat, termed Li-Qi in Chinese. After invading the human body, Li-Qi enters the lung and induces stagnation of Lung-Qi (vital essence of the lung, governing breathing and qi of the body), then to breathing difficulties, phlegm-heat accumulation and blockage, and finally bring out the dead Yin and the dead Yang. According to TCM treatment philosophy, dampness should be eliminated first, then heat should be cleared away, to restore normal bodily function (Wang et al., 2020a; Wu et al., 2021a). Its pathogenesis is characterized as “wet, heat, poison, stasis, deficiency,” accompanied by lesions manifesting mostly in the lung, spleen and stomach. TCM has accumulated much experience in dealing with this sort of disease over the centuries. In 2020, guidance issued by Taiwan’s Ministry of Health and Welfare Department of Disease Control, “New Coronavirus Disease (New Coronary Pneumonia; NCP) TCM Consultation and Staged Treatment Guidelines,” was intended to slow the development of COVID-19, block its progression to severe disease, and accelerate recovery, while simultaneously reducing disease recurrence, as well as the sequelae of severe pulmonary fibrosis and other complications (George et al., 2020). The existing literature has reviewed COVID-19 etiology, pathogenesis and syndrome characteristics as according to TCM theory, with documented evidence of therapeutic applications (Yang et al., 2020a; Qiu et al., 2020; Wang and Yang, 2021b). In contrast, our review focuses mainly on the use of TCM prescriptions and active ingredients of NRICM101, employing a literature-mining approach to analyze experimental research and clinical applications.

### 1.1 Traditional Chinese medicine theories of infectious disease

TCM has accumulated a wealth of experience regarding infectious disease. According to TCM theory, this epidemic belongs to the category of “pestilence,” with the pathological characteristics of “dampness, heat, toxin, deficiency, and stasis.” Two thousand years ago, TCM writings first documented the “Yi Bing” concept; an infective disease that differed from the common cold, with the capacity of easily becoming an epidemic. Much later, the Han Dynasty elaborated upon the dialectical relationship and treatment theory of exopathogenic wind-heat diseases (Du et al., 2020). Chinese medicine refers to wind-cold and wind-heat. Wind, cold, and heat are three common pathogens that can enter the human body from the outside. Chills, stiffness (especially in the neck), headache, and white or clear phlegm are signs of wind-chill; a sore throat, feeling warm and/or agitated (with or without fever), yellow or green phlegm, and aversion to heat are some of the signs of wind-heat (Qi and Tang, 2021). Many Chinese herbal formulas have emerged from these theoretical foundations. TCM theories relating to infectious diseases were further developed during the Tang, Song and Yuan dynasties. By the 17th century, China had published the world’s first systematic study of acute epidemic disease in the Treatise on Exogenous Febrile Disease, detailing the etiology, pathogenesis, syndrome and treatment of plague (Xu et al., 2019; Luo et al., 2020). A critical feature of TCM is that this system of medicine is able to apply the same method of treatment for patients afflicted with different diseases, who share the same syndrome. Arguably, the theoretical foundations of TCM mean that this way of treatment is an effective treatment for NCP.

Most TCM prescriptions for COVID-19 are based on heat-clearing and detoxification (Yan et al., 2020). Based on TCM theory, COVID-19 is a “warm-damp epidemic,” with a pathogenesis that is characterized by the external invasion of epidemic toxins and evil affecting the lungs and stomach. Patients with symptoms such as fever due to warm-damp epidemic poisoning should be treated with substances that clear heat, detoxify and dredge the collaterals, dispel cold and dehumidify, so the appropriate TCM formulas are those that clear heat, detoxify dampness and relieve asthma as the core treatment method. The pathogenesis of warm-damp epidemic poisoning is mostly caused by sitting and lying on the ground when the climate is humid. The spleen can transport water and dampness, which means that the spleen likes warm and dry. If the spleen is healthy, the function of transporting water and dampness is normal, and dampness does not easily cause disease. It may be necessary to take into account changes in syndromes, treat the symptoms, and increase or decrease treatment prescriptions over time.

### 1.2 Chinese medicine treatment

Importantly, TCM has proven effective against coronavirus pneumonia caused by severe acute respiratory syndrome (SARS) and Middle East Respiratory Syndrome Coronavirus [MERS-CoV] (Zhang et al., 2020). An early TCM intervention reduces the risk of morbidity and thus mortality (Qiu et al., 2020). With regard to the outbreak of COVID-19, an integrated treatment regimen of Chinese and Western medicine has been associated with significant improvements in symptoms and a shorter disease course, superior control of fever, faster clearance of chest infections and other symptoms, and a significant factor in the promotion of recovery (Yang et al., 2020c; Xing and Liu, 2021). Most TCM prescriptions used in the clinic are based on clearing heat and detoxifying, with remarkable therapeutic effects (Yang et al., 2020c). Heat-clearing and detoxifying Chinese medicines mainly include forsythia, honeysuckle, dandelion, *Houttuynia cordata* Thumb., purslane, *Isatis tinctoria* subsp. tinctoria, *Oldenlandia herbacea* (L.) Roxb., *Andrographis paniculata* (Burm.f.) Nees and lobelia (Yan et al., 2020). Heat-clearing and detoxifying drugs are used in cases of plague, sore throat, diarrhea due to heat poisoning, cancer, scalding, and epidemic fever (Yan et al., 2020). Our review of recent literature concerning Chinese medicine treatment and clinical research in infectious diseases revealed several prescriptions that are detailed below, all of which exhibit good preventive and control measures, as well as high cure rates. Among several antivirals that have demonstrated the ability to improve and eliminate symptoms of COVID-19 (Ren et al., 2020). Although remdesivir has shown potent antiviral activities, more efficacy assessments are urgently warranted in clinical trials (Beigel, et al., 2020; WHO solidarity trial consortium, 2022). Effective broad-spectrum antiviral drugs are rare because viruses are more diverse than bacteria. Antiviral drugs must enter infected cells and work, often resulting in a number of side effects (Wang and Yang, 2020; Wang and Yang, 2021b; Wang and Yang, 2022). TCM formulas are compound prescriptions that are associated with multi-target effects such as antiviral activities, regulation of immunity, and reduction of the inflammatory response, thereby improving clinical efficacy. TCM formulas that have been adopted in the treatment of mild to moderate COVID-19 cases include Qing-Fei-Pai-Du-Tang, Jinhua Qinggan Capsule, Jing Si Herbal Drink (JSHD) and Taiwan Qingguan Yihau (NRICM101) (Table 1).

**TABLE 1:** Chinese herbal products for treatment of COVID-19.

Name	Number of herbs	Composition	Original	Products	Research mode	Treatment properties	Reference
Qing-Fei-Pai-Du-Tang (QFPDT)	21	*Ephedra sinica* Stapf, *Glycyrrhiza uralensis* Fisch. ex DC., Apricot kernel L., Gypsum Fibrosum, Cinnamomum cassia Presl., *Alisma plantago-aquatica* subsp. orientale (Sam.) Sam., *Polyporus umbellatus*, *Atractylodes macrocephala* Koidz., Poria cocos (Schwein.) F.A. Wolf, *Bupleurum chinense* DC., *Scutellaria baicalensis* Georgi, *Pinellia ternata* (Thunb.) Makino, *Zingiber officinale* Roscoe, *Aster tataricus* L.f., *Tussilago farfara* L., Iris domestica (L.) Goldblatt & Mabb., *Asarum sieboldii* Miq., *Dioscorea polystachya* Turcz., *Citrus trifoliata* L., *Citrus aurantium* L., *Pogostemon cablin* (Blanco) Benth.	Ma Xing Shi Gan decoction, She Gan Ma Huang decoction (SGMH), Xiao Chai Hu, and Wu Ling San	OTC drug	Clinical trials, cell model	Antiviral, anti-inflammatory, antipyretic activity	Mehrbod et al. (2020), Zhong et al. (2020), Liu et al. (2021b)
Lianhuaqingwen Capsule (LHC)	13	*Forsythia suspensa* (Thunb.) Vahl, *Ephedra sinica* Stapf, *Lonicera japonica* Thunb., *Isatis tinctoria* subsp. tinctoria, *Mentha canadensis* L., *Dryopteris crassirhizoma* Nakai, *Rhodiola rosea* L., Gypsum Fibrosum, *Pogostemon cablin* (Blanco) Benth., *Rheum palmatum* L., *Houttuynia cordata* Thunb., *Glycyrrhiza uralensis* Fisch. ex DC., *Prunus sibirica* L.,	Maxing Shigan Tang and Yinqiao San	OTC drug	Clinical observation study, cell model	Antiviral, anti-inflammatory activity	Dong et al. (2014), Ding et al. (2017), Hu et al. (2021)
Jing Si herbal drink (JSHD)	8	*Artemisia argyi* H. Lév. and Vaniot, *Ohwia caudata* (Thunb.) H. Ohashi, *Ophiopogon japonicus* (Thunb.) Ker Gawl., *Perilla frutescens* (L.) Britton, *Houttuynia cordata* Thunb., *Platycodon grandiflorus* (Jacq.) A.DC., *Glycyrrhiza uralensis* Fisch. ex DC., *Chrysanthemum* × *morifolium* (Ramat.) Hemsl.		Food supplement	Clinical observation study, cell model	Antiviral, anti-inflammatory, antioxidative, antithrombotic activity	Lu et al. (2021), Shibu et al. (2022)
Taiwan Qingguan No. 1 (NRICM101)	10	*Scutellaria baicalensis* Georgi, *Houttuynia cordata* Thunb., Morus alba L., *Saposhnikovia divaricata* (Turcz. ex Ledeb.) Schischk., *Trichosanthes kirilowii* Maxim., *Isatis tinctoria* subsp. tinctoria, *Glycyrrhiza uralensis* Fisch. ex DC., *Magnolia officinalis* Rehder and E.H. Wilson, *Mentha canadensis* L., *Nepeta tenuifolia* Benth.	Jing Fangbaidu Powder	Prescription drug	Clinical observation study, cell model	Antiviral, anti-inflammatory activity	Tsai et al. (2021)

#### 1.2.1 Qing-Fei-Pai-Du-Tang

QFPDT formula is used in the treatment of NCP. This formula combines the four classics of Xiao Chai Hu Tang, Shegan Ma Huang Tang, Maxing Shigan Tang and Wuling San (Liu et al., 2021c). As this prescription dispels cold, clears heat and reduces dryness, it can effectively relieve several major symptoms associated with NCP, and has a clear curative effect in mild and severe infections. The main target organ of QFPDT is the lung; the second is the spleen. QFPDT helps to balance the immune system and eliminate inflammation by regulating ACE2 and protein co-expression, as well as signaling pathways implicated in disease development (Mehrbod et al., 2020). It is speculated that this formula can behave as an antiviral agent because of its ability to target ribosomal proteins found in viral replication and inhibit interactions with viral mRNA translation and other proteins (Zhong et al., 2020; Liu et al., 2021b). Thus, the antiviral effects of QFPDT are achieved mainly through different mechanisms: 1) Through direct action upon viral replication and autophagy; 2) Regulation of host pathways such as Toll-like receptors (TLRs), RIG-1-like helicases (RLH), AMP-activated protein kinase (AMPK), phosphatidyl inositol-3-kinase/protein kinase B (PI3K/AKT), and extracellular regulated kinase 1/2/mitogen-activated protein kinase (ERK/MAPK) signaling; 3) Promotion of the human defense system through T-cell and B cell functions; and 4) By enhancing the free radical scavenging activity of superoxide dismutase, catalase and glutathione peroxidase (Zhong et al., 2020). According to the literature, Ephedrae Herba and Glycyrrhizae Radix are considered to be very important in the treatment of COVID-19. Glycyrrhizae Radix is the most commonly prescribed treatment for respiratory diseases and the prescription for dyspnea is usually a combination of Ephedrae Herba and Glycyrrhizae Radix (Fu et al., 2013). Interestingly, the Ma Xing Shi Gan decoction, which is widely used in the clinical treatment of COVID-19, consists of ephedra and licorice. QFPDT requires further *in vivo* studies on the synergy and metabolism of active compounds in order to more comprehensively explain the mechanism of action and provide new ideas and evidence for further research on the use of QFPDT in the treatment of COVID-19.

#### 1.2.2 Lianhuaqingwen capsule

LHC contains 13 herbs within two TCM prescriptions: Maxing Shigan Tang and Yinqiao San. Maxing Shigan Tang was first referenced by Shanghan Lun, a book dating back to the Han Dynasty, as a treatment for febrile diseases. Bronchitis, pneumonia and early-stage measles infections have all been treated with Maxing Shigan Tang (Ding et al., 2017). Yinqiao San is described in the Qing Dynasty TCM monograph Wenbing Tiaobian as a treatment for “Warm disease” with symptoms of fever, thirst and headache. LHC has proven effective in the treatment of seasonal influenza. Randomized clinical trial evidence has demonstrated significant superiority with LHC over oseltamivir in patients with mild H1N1 infection, with LHC more effectively alleviating fever, cough, sore throat and fatigue, accompanied by shorter durations of illness and viral shedding (Dong et al., 2014). In another clinical trial involving patients with mild H1N1 infection found that LHC provided better symptom relief than oseltamivir, but similar viral clearance rates (Hu et al., 2022). LHC has been widely used in China over the last decade, providing curative evidence in various infections, for example, acute bronchitis, asthma, and chronic obstructive pulmonary disease (COPD) (Ding et al., 2017). LHC has broad-spectrum antiviral, antibacterial and anti-inflammatory functions. The main functions of LHC include the inhibition of virus binding to host cells, inhibiting virus replication and release, reducing the expression levels of chemokines/cytokines (tumor necrosis factor alpha [TNF-α], interleukin [IL]-6, monocyte chemoattractant protein-1 [MCP-1] and C-X-C motif chemokine ligand 10 [CXCL10]), enhancing the immune system and improving symptoms caused by different diseases (Shen and Yin, 2021). In a clinical trial, patients with COVID-19 infection were randomly assigned to receive usual care alone (controls) or in combination with LHC for 14 days (Hu et al., 2022). The recovery rate of the LHC treatment group was significantly higher than that of the control group and no serious adverse events were reported. LHC can be considered to improve clinical symptoms of COVID-19 (Hu et al., 2022). Most LHC adverse reactions occur after the first dose and include gastrointestinal discomfort (73.9%) and skin discomfort (9.6%), experienced largely as nausea, vomiting, diarrhea and rash (Shen and Yin, 2021). Adverse reactions are mostly mild, and monitoring should be strengthened to standardize drug instructions to ensure patient safety. LHC exhibits a broad spectrum of effects on a series of influenza viruses by interfering with both viral and host reactions (Li et al., 2020). For now, although LHC significantly relieves the clinical symptoms of COVID-19 infection, the underlying mechanism of antiviral effects in coronavirus infection, especially SARS-CoV-2, remains elusive.

#### 1.2.3 Jing Si herbal drink

JSHD, an eight-herb formula developed in Taiwan by Tzu Chi University and Tzu Chi Hospital, has received Ministry of Health and Welfare approval for use as an adjuvant therapeutic for COVID-19 infection. Therapeutic justification for this formula draws upon experience from its treatment of SARS cases in 2003. JSHD contains Yu Jen Cao (*Anisomeles indica* (L.) Kuntze), Ai Ye (*Artemisia argyi* H. Lév. and Vaniot), Ju Hua (Chrysanthemum × morifolium (Ramat.) Hemsl.), Gan Cao (*Glycyrrhiza uralensis* Fisch. ex DC.), Yu Xing Cao (*Cynoglossum austroafricanum* Hilliard and B.L. Burtt), Mai Men Dong (Ophiopogon japonicus (Thunb.) Ker Gawl.), Zi Su Ye (*Perilla frutescens* (L.) Britton) and Jie Geng (Platycodon grandiflorus (Jacq.) A.DC.). Clinical evidence continues to grow in support of JSHD as an adjuvant treatment in COVID-19 infection (Shibu et al., 2022). The therapeutic efficacy of JSHD in COVID-19 infection is supported by antiviral activity (blocks ACE2, TMPRSS2 and 3CLpro activities), antioxidative (increases SOD activity) and anti-inflammatory activities (decreases iNOS expression and NF-κB phosphorylation; inhibits the production of NO, TNF-α and IL-6), the ability to suppress immune system overactivation (by reducing macrophages, neutrophils, and white blood cells), and attenuate organ injuries (Lu et al., 2021). JSHD is a potential multi-target drug candidate for the inhibition and treatment of COVID-19, but the current study does not evaluate the interactions of JSHD Chinese herbal components in different pathways or drugs, and no relevant preclinical or clinical literature exists on JSHD. This formula can be considered as an adjunctive therapy for patients with COVID-19. Further rigorously designed studies are needed to prove the efficacy and safety of JSHD against COVID-19 infection.

#### 1.2.4 Taiwan Chingguan Yihau (NRICM101)

NRICM101 was developed in 2020 in the Republic of China by the National Institute of Traditional Chinese Medicine to relieve mild and severe cases of infectious pneumonia. NRICM101 is derived from increases or decreases to the dosage of “Mild Symptoms Decoction Pieces” detailed in the National Institute of Traditional Chinese Medicine Guidelines for Clinical Staged Treatment of Novel Coronavirus Diseases. NRICM101 has undergone clinical testing.

NRICM101 is based on “Jing Fangbaidu Powder” described in the Ming Dynasty’s Prescriptions for Taking Lives. The original recipe consists of Nepeta, Fangfeng, Bupleurum, Poria, Platycodon, Chuanxiong, Qianghuo, Duhuo, Citrus aurantium, Licorice, Ginger, and other medicinal materials. Wu Youke’s Plague Theory and Dai Tianzhang’s Guang Plague Theory proposed that “Jing Fangbaidu San” is a useful formula for those who want to sweat and cool down. It is important to relieve the evil heat of the epidemic, and the evil heat must come to an end. When the formulas are placed on the surface of the muscle, if there is no sweat, there will be no way out for evil, so the sweat method is an appropriate method for treating epidemics. Because “disease enters the lung and turns heat” is the main clinical manifestation of COVID-19, the prescription is adjusted: the patient’s condition becomes more like diffuse pneumonia, exhibiting a syndrome of lung heat and phlegm. Peppermint and mulberry leaves are the main constituents; *Scutellaria baicalensis* Georgi, *I. tinctoria* subsp, tinctoria and *H. cordata* Thunb. are used as minister medicines for clearing heat, dispelling lung and detoxification, while Quangualou widens the chest and loosens phlegm, and magnolia reduces qi and relieves asthma. Licorice is used to temper the entire formula (Tsai et al., 2021). NRICM101 was able to obtain sales licenses in Europe and the United States, thanks to the deletion of medicinal materials prohibited by foreign laws and regulations, such as *Ephedra sinica* Stapf, *Asarum sieboldii* Miq. (asarum) and *Gypsum Fibrosum* (gypsum), which allowed NRICM101 to enter the global anti-epidemic drug market; on the other hand, QFPDT contains ephedra, so it cannot be sold in the United States. In addition, gypsum is very effective in treating inflammation, because it is a mineral, it affects quality control, and finally decided to delete it. During the spread of the international new crown pneumonia epidemic, NRICM101 have been sold in the United States, Singapore, Australia, Canada, the United Kingdom, France, Belgium, Luxembourg, South Africa, Thailand and the Philippines and other countries for clinicians and the public to use correctly.

Research into NRICM101 has confirmed multiple target mechanisms, including blocking the COVID-19 spike protein receptor-binding domain from binding with the ACE2 receptor of the host cell and inhibiting virus infections, inhibiting 3CL^pro^ activity and blocking viral replication by the host cell, inhibiting alveolar macrophage secretion of proinflammatory cytokines (TNF-α and IL-6), regulating the generated inflammatory storm, reducing lung damage and reducing the development of pulmonary fibrosis (Tsai et al., 2021). More investigation is needed to explore possible effects and underlying mechanisms of NRICM101, and to determine the optimal composition of herbal ingredients to maximize the effectiveness of this formula. While the ability of NRICM101 to prevent disease progression requires further validation, our experience in Taiwan suggests a multi-targeting and potentially safe, efficacious new drug candidate.

### 1.3 Possible therapeutic effects of the NRICM101 herbs in COVID-19 treatment

NRICM101 consists of 10 herbs (Table 2). This review discusses the detailed mechanisms of NRICM101 as an adjuvant COVID-19 treatment for future clinical use and experimental reference. The relevant research and mechanisms of all NRICM101 compounds or their active ingredients are described in detail below and summarized in Table 3.

**TABLE 2:** Overview of TNRICM101 ingredients and the therapeutic efficacies of each ingredient Lin et al. (2007).

Chinese name	English/Latin name	Family	Species	Dosage (g)	Prescription functions
Huangqin	Scutellaria/*Scutellaria baicalensis* Georgi	Lamiaceae	Scutellaria	18.8	Clears Shangjiao wind-heat, antibacterial and antiviral activities
Yuxingcao	Fishwort/*Houttuynia cordata* Thunb.	Saururaceae	Houttuynia	18.8	Bactericidal antiviral cough
Gualou	Trichosanthes root/*Trichosanthes kirilowii* Maxim.	Cucurbitaceae	Trichosanthes	18.8	Removes hot phlegm and clears lung heat to improve inflammation of the lungs
Banlangen	Radix isatidis/*Isatis tinctoria* subsp. tinctoria	Cruciferous	Isatis	18.8	Antibacterial, antiviral effects and reduces the number of viruses
Houpo	Officinal Magnolia Bark/*Magnolia officinalis* Rehder and E.H. Wilson	Magnoliaceae	Magnolia	11.3	Can widen and calm the breath
Bohe	Mint/Mentha canadensis L.	Lamiaceae	Mentha	11.3	Relieves cough, resolves phlegm, clears wind and heat, and relieves muscle soreness
Jingjie	Fineleaf Schizonepeta Herb/*Nepeta tenuifolia* Benth.	Lamiaceae	Schizonepeta	11.3	Dissipates wind-heat, eliminates muscle soreness and reduces fever
Sang Ye	Mulberry Leaf/Morus alba L.	Moraceae Gaudich	Morus Linn	11.3	Enters the lung and liver meridian, clears wind and heat, moistens the lungs and relieves cough, clears the liver and improves eyesight
Fangfeng	Siler/*Saposhnikovia divaricata* (Turcz. ex Ledeb.) Schischk.	Apiaceae	Saposhnikovia	7.5	Dissipates wind-heat, eliminates muscle soreness and reduces fever
Gan-cao	Licorice/*Glycyrrhiza uralensis* Fisch. ex DC.	Leguminosae	Glycyrrhiza L.	7.5	Expels phlegm and relieves cough, clears heat and detoxifies, protects the stomach and intestines

**TABLE 3:** The potential efficacy of the herbs of Taiwan Chingguan Yihau (NRICM101) used as adjunctive therapies in COVID-19 infection.

Ingredient	Model	Active ingredients	Mechanism	Reference
*Scutellaria baicalensis* Georgi	C6/36 mosquito cell line	Aqueous extract	Antiviral activities in ZIKA22, H1N123, HIV24, and DENV25	Liu et al. (2021a)
	Vero cells	Baicalin, baicalein wogonin	Inhibit replication of SARS-CoV-2 and its 3C-like protease	Zhi et al. (2019)
	RAW264.7 cell and animal model	Aqueous extract	Reduced iNOS, Cox-2, TNF-α and IL-1β levels, and also NF-κB, MAPK phosphorylation	Yoon et al. (2009)
*Houttuynia cordata* Thunb.	HeLa 229 cells	Aqueous extracts	Blocked herpes simplex virus (HSV) infection through inhibition of NF-κB activation	Muluye et al. (2014)
	Mouse liver epithelial cell line	Quercetin, quercetrin, cinanserin	Antiviral activities in murine coronavirus and dengue virus infections	Shingnaisui et al. (2018)
	Mouse peritoneal macrophages and animal model	Volatile oil	Suppressed NO, iNOS and TNF-α	Li et al. (2011)
*Morus alba* L.	HepG 2.2.15 cell line	Mulberrofuran G, isomulberrofuran G	Anti-hepatitis B virus activity	Jan et al. (2021a)
	Vero cells	phenolic compounds	Anti-herpes simplex virus 2	Kim and Chung, (2018)
	Molecular docking		Anti-TMPRSS2 activity	Shakya et al. (2022)
	RAW264.7 cells	Sanggenon B, albanol B and sanggenon D	Reduced production of pro-inflammatory cytokines and decreased expression of iNOS and COX-2	Wu et al. (2020)
*Saposhnikovia divaricata* (Turcz. ex Ledeb.) Schischk.	Vero cells	Methanol extract	Inhibited the porcine epidemic diarrhea virus (PEDV) inhibitor	Yang et al. (2015)
	RAW 264.7 cells	70% ethanol	Inhibited the production of NO, PGE2, TNF-α, and IL-6	Chun et al. (2016)
*Trichosanthes kirilowii* Maxim.	MCF7, SK-BR3, and MDA-MB-231 breast cancer cells	Trichosanthes kirilowii Ethanol Extract and Cucurbitacin D	blocked tumor cell proliferation and induced apoptosis	Kim et al. (2013), Ku et al. (2015)
	RAW264.7 cells	Trichosanhemiketal A and B	Suppressed NO, iNOS and COX-2	Ha et al. (2019)
	H9 and C8166 cells	Trichobitacin	Anti-HIV-1 activity	Zheng et al. (2000)
*Isatis tinctoria* subsp. tinctoria	HepG2 cells	Polysaccharide.	Anti-hepatitis B virus	Zheng et al. (2000)
	Vero cells	Indigo, sinigrin, aloe emodin, hesperetin	Anti-SARS coronavirus 3C-like protease effects	Lin et al. (2005)
	Human epidermal keratinocytes and mice	Petroleum ether extract	Inhibition of IL-6, IL-33 and mast cell degranulation	Lotts et al. (2020)
*Glycyrrhiza uralensis* Fisch. ex DC.	Huh7.5 cells	Methanol extract, glycycoumarin, glycyrin, glycyrol and liquiritigenin isolated, isoliquiritigenin, licochalcone A and glabridin	Anti-hepatitis C virus	Sharifi-Rad et al. (2021)
	Vero cell	glycyrrhizin	Anti-SARS virus	Cinatl et al. (2003), Passali et al. (2021)
	RAW264.7 cells	Glycyrrhizin and isoliquiritigenin	Suppressed IL-6 and TNF-α production	Honda et al. (2012)
*Magnolia officinalis* Rehder and E.H.Wilson	Vero cell	H. officinalis extract, Honokiol	Anti-human norovirus surrogates, MNV and FCV	Poivre and Duez, (2017)
	A549 cell	Honokiol	Blocked furin-like activity and SARS-CoV-2 virus	Tanikawa et al. (2022)
	RAW264.7 cells	Magnolol	Inhibited the expression of TNF-α, IL-6 and IL-1β, reduced the expression of the NF-κB, TLR4 and MAPK pathways	Zhang et al. (2017)
	Mice	Polyphenol-rich extract	Reduced serum levels of NO, IL-6 and TNF-α in influenza virus-induced pneumonia	Lin et al. (2007), Jeong et al. (2021)
*Mentha canadensis* L.	RAW264.7 cells	Phenolic Fraction	Inhibited the expression of iNOS, TNF-α, IL-1β, and IL-6 Reduced the expression of NF-κB and MAPK pathways	Bodalska et al. (2019)
	Vero E6 cells	Aqueous extracts	Blocked virus 3CL protease and the RNA-dependent RNA polymerase	(Jan et al., 2021b)
*Nepeta tenuifolia* Benth.	HG23 cells	80% methanol	Inhibited norovirus replication through the induction of antiviral interferon production during virus replication	Lin et al. (2018)
	macrophages	Ethanol extract	Decreased COX-2 and prostaglandin E2 levels, and iNOS-mediated NO production	Jeon et al. (2019)
			Reduced the expression of NF-κB and MAPK pathways	

#### 1.3.1 Scutellaria root (*S. baicalensis* Georgi)

TCM use of *S. baicalensis* includes purposes of heat clearing, fire purging, detoxification, and hemostasis. Evidence supports anti-tumour, antiviral, antimicrobial, and anti-inflammatory activities associated with *S. baicalensis* (Zhao et al., 2016). *S. baicalensis* extracts have shown broad-spectrum antiviral activities against ZIKA, H1N1, HIV, and DENV viruses (Liu et al., 2021a). Most *S. baicalensis* ingredients are flavonoids (baicalin, baicalein, and wogonin) that reportedly inhibit *in vitro* replication of SARS-CoV-2 and 3C^pro^ (Wang and Yang, 2021c). Flavonoid-enriched *S. baicalensis* extract significantly lowers nitric oxide (NO) production and levels of TNF-α, IL-6, and MCP-1 expression (Zhi et al., 2019). In lipopolysaccharide (LPS)-stimulated RAW 264.7 macrophages, *S. baicalensis* water extract significantly inhibits NO production, interleukins 3, 6, 10, 12p40 and 17, interferon-inducible protein-10, vascular endothelial growth factor (VEGF) and keratinocyte-derived chemokines (Yoon et al., 2009).

#### 1.3.2 Heartleaf Houttuynia (*H. cordata* Thunb.)

The perennial medicinal herb *H. cordata*, often called Yu-Xing-Cao in Chinese, is widely distributed throughout East and South Asia. Traditional uses of *H. cordata* include the treatment of pneumonia, bronchitis, and chronic obstructive respiratory disease. Modern pharmacology has revealed that *H. cordata* has antiviral, anti-inflammatory, and antioxidative activities (Muluye et al., 2014). *H. cordata* has shown promising *in vitro* activity against envelope viruses, including herpes simplex virus 1 (HSV-1), influenza virus, and human immunodeficiency virus type 1 (HIV-1) (Muluye et al., 2014).

*H. cordata* essential oil inhibits activities of cyclooxygenase-2, an important inflammatory regulator (Shingnaisui et al., 2018), while *H. cordata* aqueous extracts reduce influenza symptoms and LPS-induced lung injury. Quercitrin is an important flavonoid glycoside in *H. cordata* (Li et al., 2011), capable of inhibiting the cellular content of murine Broncho alveolar fluid in mice with LPS-induced lung injury (Wu et al., 2009). Quercitrin also decreases weight loss, viral titers and mortality in mice with influenza A/WS/33 virus infection (Huang et al., 2015; Wu et al., 2021b).

#### 1.3.3 Mulberry leaf (*Morus alba* L.)

*M. alba* L. leaves can treat headache, dizziness, cough, bronchitis, asthma, and eye infection (DeFilipps and Krupnick, 2018). Traditional Korean medicine applies *M. alba* root bark for upper respiratory diseases. European countries consider *M. alba* a “superfood,” because of the high proportion of bioactive constituents supporting health and longevity (DeFilipps and Krupnick, 2018). *M. alba* juice and seeds reportedly possess activity against influenza viruses (Jan et al., 2021a). *M. alba* aqueous extract has exhibited activity against various stages of the dengue virus replication cycle (Kim and Chung, 2018). Phenolic compounds from *M. alba* root bark, including α-acetyl-amyrin, cyclomorusin, eudraflavone B hydroperoxide, kuwanon S, leachianone G, moralbanone, mulberroside C and oxydihydromorusin, have shown anti-infective activities, particularly against HSV-1 and HSV-2 replication, which could be associated with its inhibition of HSV-1 DNA polymerase and HSV-2 protease (Maryam et al., 2020). Notably, *M. alba* extract may prevent SARS-CoV-2 cell entry by inhibiting the biological process necessary for TMPRSS2 (Shakya et al., 2022). Sanggenon B, albanol B and sanggenon D from M. alba showed inhibitory effects on NO production in LPS-stimulated RAW264.7 cells (Wu et al., 2020b).

#### 1.3.4 Saposhnikovia root (*Saposhnikovia divaricata* (Turcz. ex Ledeb.) Schischk.)

*S. divaricata*, a member of the Umbelliferae family, is a perennial herb native to northern Asia. *S. divaricata* is listed as a high-grade drug in the Chinese Materia Medica that is indicated for the treatment of vertigo, headaches, generalized aching and arthralgia associated with “wind, cold, and dampness.” *S. divaricata* root extract has inhibitory effects on the central nervous system and peptic ulcer, reduces fever, and has analgesic, anticonvulsant, antioxidant and anti-inflammatory activities (Yang et al., 2020b). Chemical research into this herb has isolated several different components including chromones, coumarins, lignans, polyacetylenes and sterols (Yang et al., 2020b). Some evidence suggests that coumarins from *S. divaricata* inhibit the activity of porcine epidemic diarrhea virus (Yang et al., 2015). Ethanol extracts of *S. divaricata* showed anti-inflammatory activity by inhibiting the production of NO, prostaglandin E2 (PGE2), TNF-α, and IL-6 in LPS-induced RAW 264.7 cells (Chun et al., 2016).

#### 1.3.5 Mongolian snakegourd fruit (*Trichosanthes kirilowii* Maxim.)

*T. kirilowii* is a perennial liana plant of the Cucurbitaceae family that is mainly found throughout East Asia. It is an important medicinal plant that is commonly used in Chinese herbalism. *T. kirilowii* tuber extract has long been used in Eastern Asian medicine to alleviate diabetes symptoms. Several types of cucurbitacins demonstrate anti-inflammatory and anticancer activities, including cucurbitacin D, which has been isolated from *T. kirilowii*. This substance induces apoptosis and suppresses tumor cell proliferation by inhibiting signal transducer and activator of transcription 3 (STAT3) and phosphorylating nuclear factor kappa B (NF-kB) (Kim et al., 2013; Ku et al., 2015). Trichosanhemiketal A and B from *T. kirilowii* inhibited inducible nitric oxide synthase (iNOS) and cyclooxygenase (COX)-2 expression (Ha et al., 2019). In addition, trichosanthin extract from *T. kirilowii* potently inhibits HIV-1 replication and exhibits antitumor activities (Zheng et al., 2000).

#### 1.3.6 Indigowoad root (*I. tinctoria* subsp. tinctoria)

*I. indigotica* root (Radix isatidis) is a member of the Cruciferae family and a native Chinese herb. *I. indigotica* root and other phenolic Chinese herbs were popular amongst TCM practitioners for controlling SARS outbreaks in 2002–2004 throughout China, Hong Kong and Taiwan. *I. indigotica* root has antiviral activity against influenza, hepatitis A and Japanese encephalitis (Chen et al., 2012). Substances found in *I. indigotica* root include indican (indoxyl-β-d-glucoside), indigo, indirubin, β-sitosterol, γ-sitosterol and sinigrin. Indigo, sinigrin, aloe emodin and hesperetin have all demonstrated anti-influenza virus effects by blocking 3CL^pro^ cleavage (Lin et al., 2005). Petroleum ether extract of *I. indigotica* reportedly inhibits the levels of IL-6 and IL-33 expression and mast cell degranulation (Lotts et al., 2020).

#### 1.3.7 Baked licorice root (*Glycyrrhiza glabra* L.)

*G. glabra* L. (Fabaceae family), commonly known as licorice, is a small perennial herb indigenous to West Asia. The *Glycyrrhiza* genus is found worldwide and contains over 30 species. Rhizomes and roots are the most important medicinal parts of licorice and are used alone or in combination with other herbs in the treatment of diverse digestive system disorders (e.g., stomach ulcers, hyperdipsia, flatulence, colic), respiratory tract disorders (e.g., asthma, coughs, sore throat, and tonsillitis), jaundice, hemorrhagic diseases, malaria, fever, epilepsy, sexual debility, paralysis, rheumatism, leucorrhea, psoriasis, and prostate cancer. This substance is also used to flavor foods and beverages (El-Saber Batiha et al., 2020).

Several active compounds are found in *G. glabra* roots, including the flavonoids glucoliquiritin, licoarylcoumarin, licopyranocoumarin, liquirtin, liquiritigenin, rhamnoliquirilin, and glycyrrhizin. The main active constituent of *G. glabra* is glycyrrhizin, capable of inhibiting virus-cell binding and beneficial in the treatment of HIV-1 and chronic hepatitis C virus (HCV) infections (Sharifi-Rad et al., 2021). Glycyrrhizin markedly inhibits SARS viral reproduction (Cinatl et al., 2003; Passali et al., 2021). In addition, glycyrrhizin and isoliquiritigenin suppress LPS-induced activation of signaling cascades and cytokine production (Honda et al., 2012).

#### 1.3.8 Magnolia bark (*Magnolia officinalis* Rehder and E.H. Wilson)

*M. officinalis* is an important TCM herb for the treatment of a wide variety of conditions, including gastrointestinal disorders, typhoid fever, thrombotic stroke, allergies, anxiety, and nervous disturbances. Biologically active compounds found in *M. officinalis* include 4-O-methylhonokiol, honokiol, magnolol, obovatol, and other neolignan compounds. The two major metabolites, honokiol and magnolol, have wide-ranging pharmacological effects, including antitumorigenic, anti-inflammatory, antithrombotic, antioxidative, and neuroprotective activities (Honda et al., 2012). *M. officinalis* extract and Honokiol reportedly suppresses against human norovirus surrogates, murine norovirus (MNV) and feline calicivirus (FCV) *in vitro*, and in model food system (Kim et al., 2021). Honokiol inhibited the furin-like activity and SARS-CoV-2 infection (Tanikawa et al., 2022). Interestingly, high levels of NO, IL-6 and TNF-α expression in a mouse model of pneumonia were effectively lowered after treatment with polyphenol-rich extract from *M. officinalis* bark, which also provided long-term protection against reinfection (Lin et al., 2007; Zhang et al., 2017; Jeong et al., 2021).

#### 1.3.9 Peppermint herb (*Mentha canadensis* L.)

*M. canadensis*, a perennial herbaceous plant of the Lamiaceae family, is found throughout Taiwan and popularly used in food, cosmetics and medicines. In TCM, *M. canadensis* is used to treat diseases related to the nervous, respiratory, reproductive and digestive systems (He et al., 2019). Pharmacological studies of *M. canadensis* have revealed antimicrobial, anti-inflammatory, antiviral, antioxidant, antitumor, gastrointestinal protective and hepatoprotective activities (He et al., 2019). *M. canadensis* contains many volatile compounds including menthol and menthone, as well as polyphenolic acids, flavonoids, monoterpenoids and glycosides, which are speculated to be responsible for the medicinal benefits (Tafrihi et al., 2021). Amongst *M. canadensis* -related flavonoids, hesperidin and linarin are abundant active substances. Hesperidin has shown anti-inflammatory, antioxidative and antitumor activities, while linarin has anti-inflammatory and acetylcholinesterase activities (Bodalska et al., 2019). In addition, in a hamster disease model of SARS-CoV-2 infection, *M. canadensis* prophylaxis effectively prevented the disease from developing (Jan et al., 2021b).

#### 1.3.10 Nepeta tenuifolia (*Nepeta tenuifolia* Benth.)

*N. tenuifolia* belongs to the Lamiaceae family and has a long history of medicinal use in China, Taiwan, Japan and Korea. *N. tenuifolia* is used to treat symptoms of common influenza (e.g., fever, headache, virus infections and sore throat) and is supported by pharmacological evidence of anti-inflammatory (Kim and Chung, 2018), immunomodulatory, antioxidative and antipruritic activities (Lin et al., 2018; Jeon et al., 2019). The major identified constituents in this substance are (−)-pulegone, (+)-menthone and (+)-limonene. *N. tenuifolia* has been marketed for the treatment of common cold and fever in Chinese proprietary medicines (Fung and Lau, 2002).

Ten constituent herbs were identified in an HPLC fingerprint analysis of NRICM101 (Tsai et al., 2021). Baicalin is the main compound in NRICM101 (2.38 mg/ml); many other compounds that have been identified include mainly caffeoylquinic acid, quercetin, rutin, chrysin, baicalin, breviscapine, wogonin, and licorice.

### 1.4 Clinical study of the traditional Chinese medicine for the treatment of COVID-19

A randomized clinical trial of 98 confirmed cases were treated with QFPD, patients were treated for 9 days and the total effective rate was 92.09%, of which the recovery rate was 41.13%, the significant efficiency was 26.92%, and the effective rate was 24.04% (Wang, et al., 2020b). QFPDQ has a good therapeutic effect on the treatment of COVID-19 in clinical practice. In addition, when used as adjuvant therapy with western medicine, QFPD can relieve symptoms and improve pulmonary inflammation regression, showing a trend of reducing the degree of multi-organ damage in patients with COVID-19 (Xin, et al., 2020). It significantly improves the clinical symptoms of patients, reduces the adverse reactions of patients, and improves the therapeutic effect.

A randomized clinical trial on LHQW of 284 (142 each in treatment and control group) COVID-19 patients is useful to confirm the beneficial effect. In the trial, 142 patients were given LHQW for 14 consecutive days, the results manifested that the recovery rate (91.5% vs. 82.4%), the improvement rate (83.8% vs. 64.1%) and the clinical cure rate (78.9% vs. 66.2%) of the treatment group were higher than the control group (Wang et al 2020a). Time to recovery of fever (2 vs. 3 days), fatigue (3 vs. 6 days) and coughing (7 vs. 10 days) was also significantly shorter in treatment group. This trial showed that LHQW could be considered to ameliorate clinical symptoms of COVID-19 (Hu, et al., 2022).

In clinical studies, NRICM101 (100 ml) was administered three times a day, 30 min after each meal, until hospital discharge (Tsai et al., 2021). Taiwan study discharge criteria stipulated three consecutive negative test results for SARS-CoV-2 in respiratory tract samples that were collected at ≥24 h intervals (3 N). Any adverse events were recorded on a standard form. In patients given symptomatic care only, 3 N was reached at a median of 22 days after the onset of hospitalization. NRICM101 was administered to those patients who failed to show signs of improvement after a median 21.5 days in hospital; 3 N was observed at a median 9 days after intervention. However, while the evidence suggests that NRICM101 shortens the time to symptom recovery, no between-group differences were observed in the conversion rate to severe cases or viral assay findings (Tsai et al., 2021). An analysis of the literature has found that NRICM101 can reduce virus infections and replication withdrawal syndrome in patients with COVID-19 (Tsai et al., 2021). In light of the safety and effectiveness profiles, NRICM101 can be considered as capable of ameliorating clinical symptoms of COVID-19 (Tsai et al., 2021). The potential feasibility of TCM prescriptions for the treatment of COVID-19 is elucidated. More experimental and clinical research is needed to prove the safety and effectiveness of this formula.

## 2 Conclusion

The COVID-19 epidemic remains serious, with no available measures or drug treatments that can effectively control the spread of infection. For those in home isolation, TCM can be used to prevent and avoid severe illness. The ingredients prescribed are mainly polyphenols, which are used to avoid infection and boost autoimmunity (Ali et al., 2013). Prescriptions for COVID-19 prevention based on the “natural factor” theory were used to maintain life qi (Liu et al. 2022). Modern pharmacological studies have shown that TCM ingredients have the functions of clearing away heat and detoxification, removing moisture from the surface and inhibiting viruses. Much evidence shows that TCM can improve the symptoms of COVID-19, delay the clinical deterioration, and reduce all-cause mortality in severe cases. Different combinations of TCM compounds may improve therapeutic efficacy. There are two ways in which TCM acts as an antiviral drug. One is to directly inhibit the entry and replication of the virus, mainly to clear heat and detoxify such as honeysuckle, Radix isatidis and Scutellaria for inhibition. The second is to indirectly exert antiviral effects or inhibit virus-mediated inflammatory responses by regulating the immune function of organisms. Astragalus, licorice and mint are used as antivirals and induce interferon and immunoglobulin. TCM focuses on the overall regulation of the body. Therefore, TCM can play a multi-target role in the regulation of body systems (Zhong, et al., 2019). A single targeted drug may not be suitable for curing complex diseases such as COVID-19. Chinese medicine has good potential to complement the medical treatment of COVID-19, in part perhaps because of the multi-component and multi-target effects of Chinese medicine. Different Chinese medicines may act on the same or similar targets or pathways. However, current understandings of the mechanisms of action of TCM have mostly been generated by molecular analysis and network pharmacology-based approaches. *In vitro* cellular assays and *in vivo* animal studies are required to confirm these predicted mechanisms. In addition, many clinical studies and statistical methods still have the safety and efficacy of TCM in the treatment of new coronavirus infection should be carefully evaluated. It remains necessary to elucidate the mechanisms of action of TCM that ameliorate symptoms of COVID-19. Further evaluations are needed to explore the clinical efficacy of TCM against COVID-19, potential molecular mechanisms that improve multiple organ damage, and use various modern scientific techniques to determine the material basis of possible drugs. It is hoped that this review can provide a reference and basis for the development of new drugs for the prevention and treatment of viral infectious diseases such as COVID-19.

NRICM101 is an herbal formula that has been developed according to TCM theory and preclinical experiments. Mechanism of action studies involving NRICM101 have shown that NRICM101 exerts potential therapeutic effects by comprehensively regulating multiple targets *via* multichannel synergistic mechanisms, providing a theoretical basis for further empirical research and drug development. However, despite the promise of in-depth research into pathogenic targets of COVID-19, the global outbreak continues to worsen. It is therefore hoped that NRICM101 may be used in addition to conventional drug therapy for treating COVID-19 infection. More clinical studies are needed to explore NRICM101-associated efficacy and safety in COVID-19 infection.
